# Auto-Configuration in Wireless Sensor Networks: A Review

**DOI:** 10.3390/s19194281

**Published:** 2019-10-02

**Authors:** Ngoc-Thanh Dinh, Younghan Kim

**Affiliations:** School of Electronic Engineering Soongsil University, Sangdo-Dong, Dongjak-Gu, Seoul 06978, Korea

**Keywords:** wireless sensor network, auto configuration

## Abstract

Wireless sensor network (WSN) studies have been carried out for multiple years. At this stage, many real WSNs have been deployed. Therefore, configuration and updating are critical issues. In this paper, we discuss the issues of configuring and updating a wireless sensor network (WSN). Due to a large number of sensor nodes, in addition to the limited resources of each node, manual configuring turns out to be impossible. Therefore, various auto-configuration approaches have been proposed to address the above challenges. In this survey, we present a comprehensive review of auto-configuration mechanisms with the taxonomy of classifications of the existing studies. For each category, we discuss and compare the advantages and disadvantages of related schemes. Lastly, future works are discussed for the remaining issues in this topic.

## 1. Introduction

Due to the cheap cost and the ease of deployment, sensor networks have been deployed in various areas, such as in home automation, building automation, urban areas, and industrial zones [1]. However, the nature of resource-constrained wireless sensor networks (WSNs) [2] require an efficient way for the network design, deployment, and configuration [3]. Moreover, WSNs are normally a large collection of resource-constrained sensors that cover a wide area, so the network configuration is challenging [4,5,6,7,8]. To cope with the above requirements, various auto-configuring schemes for WSN have been designed and optimized for efficiency and scalability.

In the time dimension, the configuration in a WSN can be divided into two stages: initial configuration [9,10,11,12,13,14,15,16,17,18,19,20,21,22,23,24,25,26,27] when WSNs are deployed and updating configuration [28,29,30,31,32,33,34,35,36,37,38,39] which occurs during the runtime of WSNs. The first one usually deals with assigning some parameters such as address [9,10,11,12,13,14,15,16,17,18], roles [19,20,21,22,23,24], and QoS (Quality of Service) parameters [25,26,27] for sensors in a newly deployed WSN in order them to interact with other nodes. The second stage is responsible for updating new requirements, applications, or software throughout the network [40,41,42,43,44,45,46,47,48,49,50].

Unfortunately, a network containing a large number of nodes also leads to difficulty in the configuring process [8]. Within the small sensor network, an administrator easily assigns addresses and roles and updates software and firmware for all nodes. However, when the number of nodes increases, the former way is unsuitable, and, thus, results in a new required approach: auto-configuration.

Auto-configuration in WSN [24] is a general name for proposals characterized by an update or configuring demand. This type of configuration is simply deployed at a sink and then automatically relayed to other nodes in a WSN. This simple mechanism achieves various advantages: it is easy-to-use, it has a quick deployment, and it is energy efficient.

Although auto-configuration is a powerful approach for deploying and updating a WSN, there were no previous surveys that provided a full understanding of auto-configuration in WSNs. Therefore, to ensure complete knowledge about recent research on auto-configuration, we have provided the taxonomy of classification in this paper. To understand the problem in detail, it is necessary to have an accurate, detailed comparison of existing solutions. This paper also presents an objective point of view regarding the advantages and disadvantages of each scheme. This would ensure, therefore, that it would be possible to discover any possible issues for future work.

The remainder of this paper is categorized as follows: Section 2 discusses the main challenges that can be presented while deploying a sensor network and in the update process. The next section provides a comprehensive taxonomy of the current solutions and analyses and comparisons of these solutions. Implications for future work will be presented in Section 4. Lastly, the final section contains the conclusion.

## 2. Research Challenges in WSN Configuration

The entire life of a WSN could be divided into the following four stages: starting, maintaining, updating, and ending. The first stage deals with how to setup the initial parameters for all nodes. The second stage solves the problem of transmitting information in an optimal way such as routing [4], maximizing network lifetime [5], coverage [6], and data aggregation [7]. The third focuses on how to efficiently deliver new update information to each of the nodes. Finally, the final stage involves shutting down all sensors. Although most of the proposals in WSNs have focused on the second stage, as the first and the third only happen in short intervals, they each play their own roles and still are important, as they contain open issues that are yet to be solved.

When a sensor is powered up, some parameters should be configured to help that node communicate with the sink and other nodes. Address [9,10,11,12,13,14,15,16,17,18], roles [19,20,21,22,23,24], and QoS-parameters [25,26,27], and other configurations [40,41,42,43,44,45,46,47,48,49,50] are parameters that must be considered in this context. In the third stage, once there is a new application or a new requirement, it should be delivered to all nodes in a WSN.

Regardless of what could be assigned for each sensor, all of nodes in a WSN should be configured with that information. Unfortunately, a WSN is usually a collection of a large number of nodes [8]. Therefore, the above-mentioned requirement turns out to be quite difficult. Besides, the sensors are energy-constrained [2]. Each of them is usually supplied by a set of batteries. This kind of resource power is enough for a short lifetime only and is difficult to recharge.

To meet with the above requirements, an administrator could consider turning on and off a sensor to manually configure each one. If a WSN has tens or hundreds of nodes, physical configuration could be possible. However, what will happen if this number reaches thousands or millions? Obviously, the above solution would be an improper option. Time constraints motivate administrators to discover new solutions for this problem.

**Figure 1:**
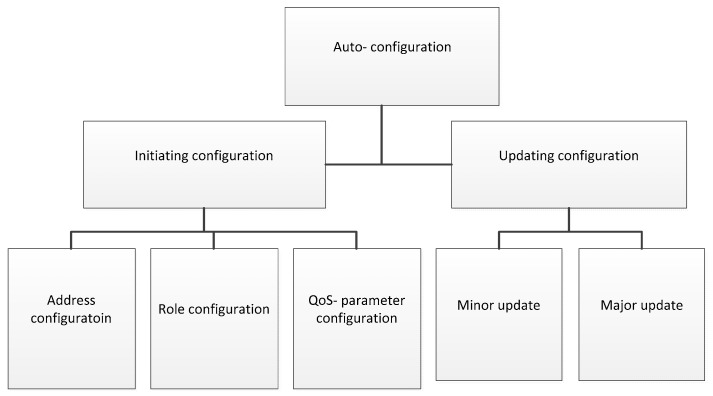
The taxonomy of the existing auto-configuration schemes in wireless sensor network (WSNs).

Therefore, auto-configuration is a good choice up until this point, as is shown in Figure 1. However, upon closer examination of each of the aforementioned problems, it becomes evident that a list of open issues exists and needs to be solved as soon as possible. One example is the issue of addressing which scope of an address should be covered: local [14] or global [10]. One issue arises when determining whether an address should attach with additional parameters, such as location information [16,17,18]. In the QoS-parameter assigning issue, which one should be configured? Furthermore, if there is more than one QoS parameter, then should there be a trade-off among them in order to achieve the best solution? In other cases, updating for a WSN must be highly reliable. In addition, rapidly detecting new methods of requirement and delivery to other nodes are other challenges for researchers in this field.

In addition, there are more and more applications of sensors in today’s world. Moreover, sensor systems are getting more complex and they are used for many high-risk security-critical purposes. Existing sensor security facilities and methodologies are relatively poor during update processes. Therefore, a methodical approach to WSN security during configuration is needed, i.e., risk management, implementation of countermeasures, vulnerability removal, and security evaluation and certification, especially in critical sensor applications like e-healthcare or military.

### Taxonomy of the Existing Auto-Configuration

Figure 1 illustrates the comprehensive categories of auto-configuration proposed for a wireless sensor network. We classify the existing schemes into two categories including initiating configuration or updating configuration, based on the stage of the lifetime of a sensor. We further divide existing works into sub-categories based on their purpose of configuration. In the initiating configuration category, we divide existing works into address configuration, role configuration, and QoS parameter configuration. In the updating configuration, we divide existing works into minor update and major update. We present the details of each approach and compare them together in the next sections.

## 3. Initiating Configuration

In this stage, sensors are powered up and begin to collect information for the initial setting. There are various configuration parameters required for a host.

### 3.1. Address Configuration

**Figure 2:**
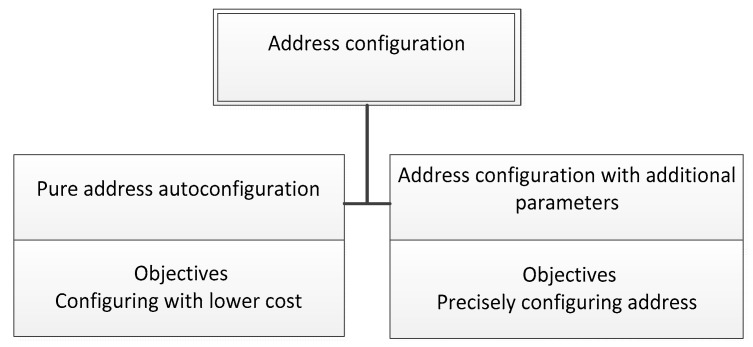
The taxonomy of the addressing configuration problems.

The traditional approach of address configuration uses centralized servers such as the DHCP server and the DNS server. Unfortunately, the centralized method actually takes advantages as if the total number of nodes is small. Having a large number of sensor nodes is a significant challenge. From an economical viewpoint, we categorized address configuration into two branches. One is a pure address configuration, which occurs when a node simply requires an address to communicate with others without any additional information. The other is address schemes with the support of some parameters such as location information. The first one allocates the address for each node with a low cost. The second one precisely assigns the address for each sensor in terms of location. However, it increases the cost of the whole system by adding more information. Our suggested taxonomy is based on the above requirements, as we have demonstrated in Figure 2.

### 3.2. Pure Address Auto-Configuration

In this category, an address is assigned to each node without concerning any additional information, such as location, in order to minimize the cost for the whole network. The process of allocating the address is as follows: first, a sink, a gateway, or a server reserves a list of possible assigning addresses. Then, these addresses are delivered to all possible nodes. This can be done by responding to the request of a sensor that has just joined the network or been automatically delivered to all nodes in the network in order to initiate all sensors. After receiving an address, the function “Duplicate Address Detection-DAD Mechanism” is used for checking for any address conflictions with other nodes. The following is the proposed mechanism. Table 1 presents a summary of each address in the auto-configuration scheme.

#### 3.2.1. Proxy-Based Address Assignment

**Figure 3:**
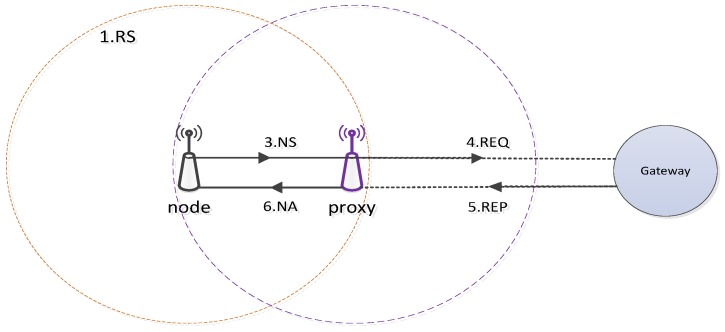
Processes of allocating IP addresses through a proxy node.

The approach [9] is an IPv6-addressing mechanism, which provides a lightweight unique 16-bit ID for each node. Figure 3 shows the main operations of this scheme.

The process of obtaining an address is divided into two steps. Once a node wants to obtain an address, it finds a proxy, which acts as an anchor by broadcasting a router solicitation message (RS) to all one-hop neighbors. After that, a suitable proxy will reply a router advertisement message (RA) with a detection address duplicate flag. This process will be repeated from the gateway to the edges of the network. Secondly, the node’s address will be auto-configured by using the network prefix and an identifier. Based on the address of the proxy, the node registers an address with a gateway by sending a neighbor solicitation message to its proxy. Then, the proxy will send a request (REQ) message to a gateway to obtain an IP. The gateway sends back a reply (REP) in the next step to the proxy. After receiving that reply message, the proxy will create and forward a neighbor advertisement packet (NA) to configure the IP address of that node.

Obviously, this mechanism is a centralized approach and requires a high cost in terms of the latency of the control message because of sending the packet to both a proxy and a gateway. However, this protocol is greatly improved by deploying the immediate address assignment scheme. Instead of sending the packet from a proxy to a gateway, in the alternative approach, the proxy only sends the packet to an address pool that has been pre-allocated in an intermediate node. The simulation result [9] indicates that by applying the new mechanism, the latency and overhead are greatly reduced. In addition, the mobility of addressing and reusing the IP address is resolved. However, the 16-bit address is only for local communication and cannot communicate with the Internet without further support.

**Figure 4:**
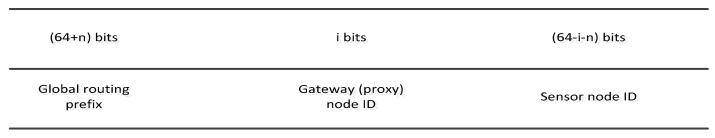
The IPv6 address structure (adapted from [10]).

Similarly, in [10], the process of acquiring an IPv6 address is similar to that of [9]. However, the authors change the structure of the IP address to support communicating with outside networks. In this case, each address consists of two parts. One is the global routing prefix, which is unique for each network. The remaining one is the interface ID, which is further divided into the gateway node ID and sensor node ID, as is illustrated in Figure 4.

Two kinds of messages are also supplemented to update the lifetime of the corresponding address entry: Live packet and Update packet.

The results in [10] also indicate that the address configuration cost is lower than that of [9]. Delay is also improved greatly in half of the time compared to that of [9]. Nevertheless, adding a global routing prefix leads to increasing headers for each packet.

Beside the advantages of having a lightweight IP address and scalability, both above proposals are presented with the challenge of having a significant delay. This is because the addressing mechanism begins with a request of a node, rather than by being broadcast from a sink, as is demonstrated in the following approaches.

#### 3.2.2. Multi-Tiers Configuration

The proposals [11,12] assign an address for all nodes quickly by starting from a sink. The address of a node is formed by the address of its parent and an additional bit. Although they quickly deploy the IP address, these mechanisms face the challenge of increasing the length of an address dramatically. Below is one of the proposals.

**Figure 5:**
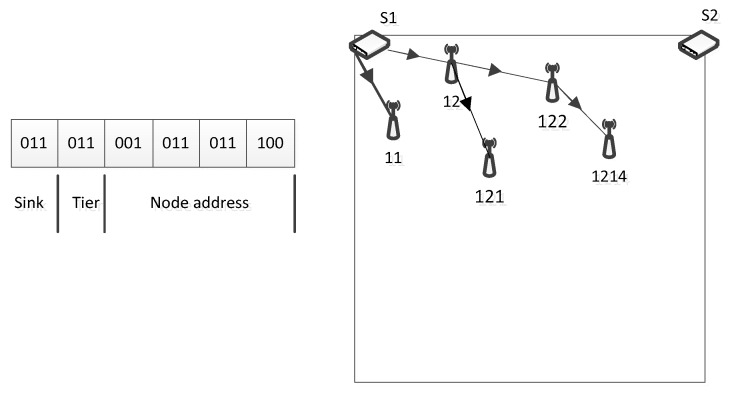
The addressing scheme and configuration using the multi-tiers configuration (adapted from [11,12]).

The proposal in [11] presents an addressing scheme that uses a multi-tier mechanism, similar to Figure 5. First, the process began from a sink by broadcasting to its neighbors a message containing the sink’s address. The neighbors will confirm to the sink that they have received the message by sending a reply message back with a unique flag. Then, the sink chooses an address for each of its children and delivers those addresses to the children. An address is formed by the sink’s address and a random sub-address. For instance, a sink whose address is 1 will provide its children with the addresses 11, 12. This process is repeated until all of the nodes are addressed.

However, rapidly increasing the length of each address results in the nature of the concept of tier and level to be applied. Within an address space, there are multiple tiers, and within a tier, there are multiple levels. The basic concept of the tier and level are presented on the left side of Figure 5. A threshold is used for determining a tier. For instance, an address that is 1-12-122-1224-1-12-122-1224-1-12-122 can be shortened as 1 (sink address) 010 (tier 2) 1-12-122 (level 3 with a specific address of 122).

Although this provides a scalable and easy addressing mechanism, this scheme did not support the duplicate address detection mechanism. Then, it cannot guarantee a confliction while a node transfers a packet. By analysis, the author proved that this mechanism can support a large number of networks with a small number of control messages.

#### 3.2.3. Cluster-Based Addressing

**Figure 6:**
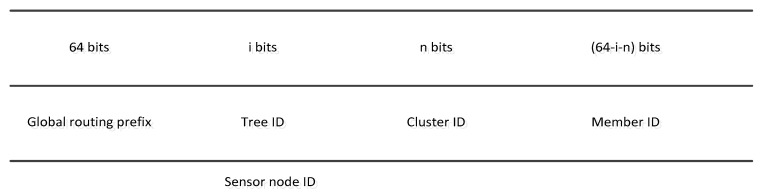
The IPv6 address structure using cluster-based addressing (adapted from [13]).

With an intention of rapidly deploying IP addresses and providing a scalable mechanism, the scheme in [13] is proposed by utilizing the clustering tree architecture. In this approach, a sensor network is managed into multiple sub-networks. Each subnet is responded by a cluster head. This addressing scheme is also divided into two phases. One is allocated to the cluster head, and one is allocated to the normal nodes. For each cluster head, a stateful IP address is delivered from the gateway to avoid DAD, while a stateless IPv6 address is assigned for each one. Additionally, once a normal node wants to acquire an address, it shows its presence by broadcasting to join a cluster tree. Then, based on the distance from the cluster head to a sink, the node will choose the nearest cluster and request an IP address. After that, the cluster head will respond via an ACK packet. This ACK message contains the assigned IPv6 and should be sent to the required node, which has the following structure demonstrated in Figure 6.

The procedure of addressing a cluster head is quite similar to that of a normal node. However, the type of broadcasting and acknowledging messages are different from those of the previous one. In addition, the cluster head also has to calculate to reach the optimal coverage.

The simulation results in [13] show that by applying the mechanism above, the address’ duplicate cost and delay are both reduced dramatically. In addition, the analysis in [13] also indicates that the scalability is high with low cost. This is beneficial for WSN, due to the presence of a large number of nodes.

#### 3.2.4. Localized Address Assignments

In other directions with the above proposals, the objective of the localized address assignment is to reduce the delay and cost for allocating an IP address of a node by using a local server. Inspired from DHCP and Passive Duplicate Address Detection (PDAD) protocol, localized addressing allocates an IP address by choosing a dedicated server. This server firstly broadcasts a round ID to all of its neighbors. Then, the neighbors send HELLO messages in response to that server in order to verify the round ID. The address of a node will be chosen randomly each time the node receives a message with a new round ID.

**Figure 7:**
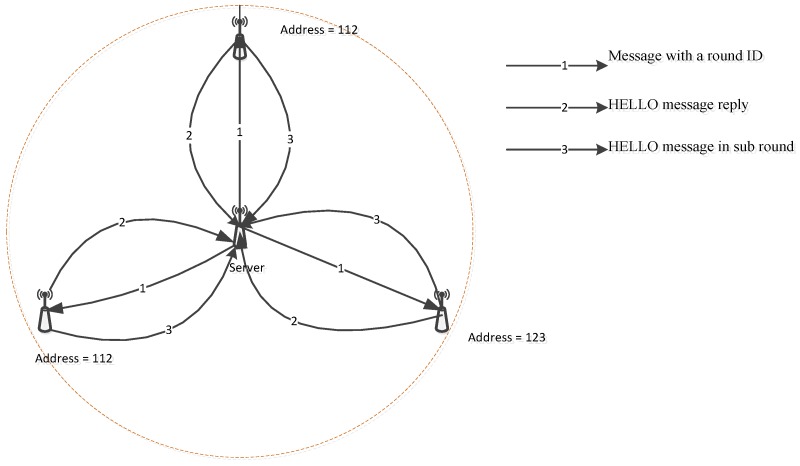
An addressing scheme and possible duplicate.

If an address is duplicated, the local server then creates a sub-round in which a sensor node attaches a randomly-selected address in HELLO message to send back to a server, as is shown in Figure 7. A new address should be generated if the former address is already available.

**Figure 8:**
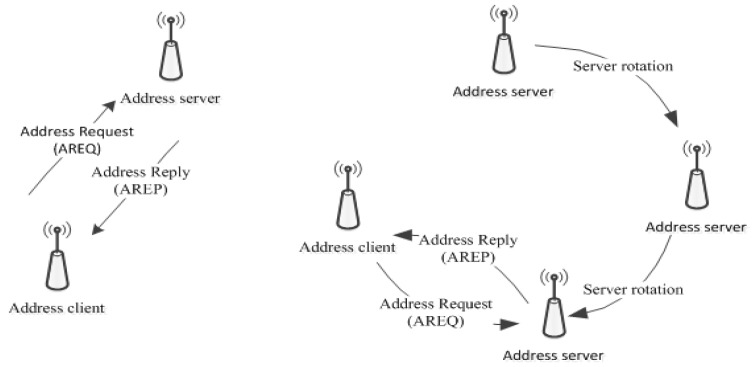
An addressing scheme and server rotation scheme (adapted from [15]).

Although this kind of mechanism provides a unique address scheme, which is useful for local communication, it is an obstacle to connect with the outside network. Additionally, the local server rapidly depletes energy in the addressing process. To cope with this challenge, another mechanism is proposed in [15] by sequentially changing local servers, as is shown in Figure 8, in which a local server acts for a short time and is elected among sensors.

### 3.3. Configuration with Additional Information

A different goal from pure address auto-configuration, configuration with additional information provides a location-precise way of assigning address for each node. An address is attached with the geographic information for efficient management and support of the upper layer application. The following approaches are listed in Table 2.

#### 3.3.1. Group-Addressing

The protocol in [16] takes advantages of cluster-based address configuration to reduce network size and enhance energy efficiency and scalability by assigning an address for a group of sensors as a representative address. In this paper, a sensor network is graphically separated in multiple sub-networks. Each sub-network is called a sensing unit, and a cluster head responds for a sensing unit.

Once the network starts up, a sink provides information to all cluster heads by broadcasting its location information. In order to obtain an address, the cluster head replies back an address request message with its location. Based on the distance between the sink and the cluster head, the required number of cluster heads and the distribution of the cluster heads follow the Voronoi diagram and is calculated at a sink. Then, a node calculates its own address relying on its position information.

To prevent the duplicate address problem, the color-address mapping is applied and is responded to by the sink node. This color-address mechanism follows the rule “if and only if the greatest distance between sensor nodes in two different clusters is beyond the communication range of the sensor node, the two clusters can be assigned the same cluster-color.” This is in order to discover the direction of a potentially occurring event. Then, each address of a sensor contains a cluster-color or color-address, distance-address, and sector-address of sensing unit. The color-address is similar to a unique address for a cluster area.

This kind of approach is both scalable and energy-efficient because of the reducing addressing area. However, an address is only for a sensing unit. If there are multiple kinds of sensors in the same area, this scheme is not suitable. In addition, the addressing mechanism is complicated because there are multiple parameters that should be calculated, such as direction and distances.

#### 3.3.2. Geometric Information Based Addressing

**Figure 9:**
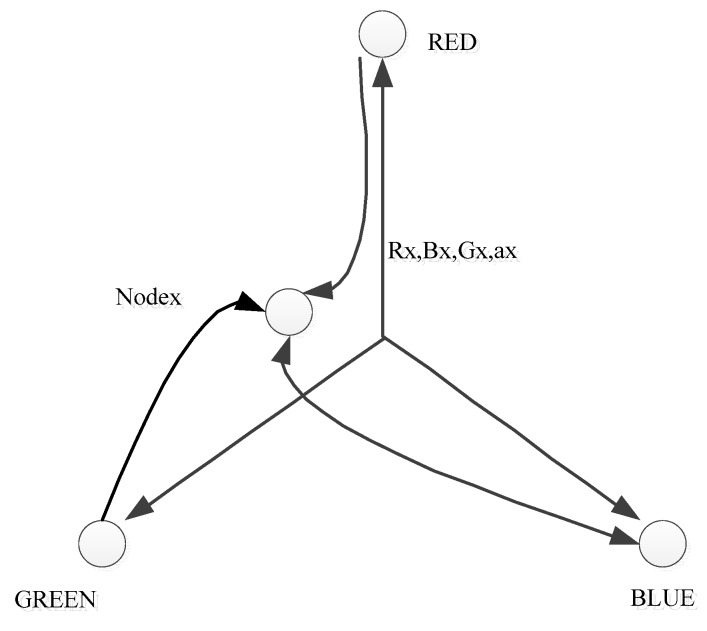
An algorithm of the three-axis method (adapted from [17]).

Contrasting from the proposal in [16], the protocol in [17] utilizes the geometric information to auto-configure 16-bit IP addresses for each node in WSNs. However, 16-bit address space also leads to increasing address-confliction among nodes in a WSN. Therefore, there should be an efficient addressing mechanism that exists to cope with this challenge. In [17], a network static deploys three nodes to become three coordinators (RED, GREEN, BLUE). A node calculates the distance from itself to the coordinators and a randomly generated number to calculate its address, which is formed by a set of values (red, green, blue, alpha), as is shown in Figure 9.

However, differencing hop distance results in the same color zone, which is defined as the monochrome zone. A monochrome is a representative of a confliction zone. In that area, there are multiple nodes with the same color. Then, if the address of a node is detected to be conflicted, it regenerates its own address and broadcasts it. The monochrome zone is expanded to avoid the recursive conflict. In addition, in order to enhance the performance of this kind of addressing, three coordinators should be used as far as possible. Although this protocol is very simple, by simulation and testing, the authors proved that it can support mobility, provide a lightweight address, and reduce the address duplication cost. However, the authors did not show any methods to protect the existence of three coordinators. If these nodes die or experience a loss of the link, this leads to a problem in configuration.

#### 3.3.3. Location-Based IP Addressing

**Figure 10:**
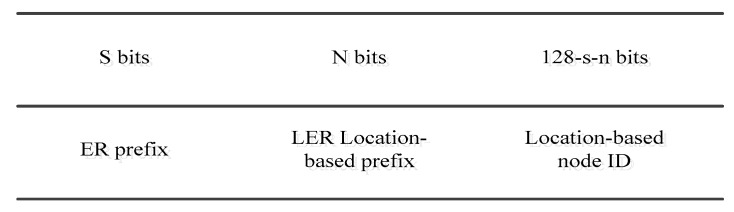
The IPv6 address structure with the location-based addressing (adapted from [18]).

To further explain the low layer information in the addressing process, the following approach uses the physical location information to generate an IP address. The network is divided into multiple sub-networks. However, it is different from the proposal in [18], as each sensor is assigned a 128-bit address. Each address consists of a prefix of subnet, location, and location-based node ID, as is shown in Figure 10.

In addition, the structure of network is as follows: an edge router is found first, at the top level. The full function devices are deployed as the local edge routers (LER), which are responsible for all nodes within their scopes. The reduced function devices are considered as normal nodes that hold the location-based node ID. In addition, this kind of protocol uses the physical location information, which will increase the deploying cost of the whole network and make it more difficult to implement.

This kind of scheme is not considered highly scalable. However, the method of deploying full function devices (FFD) and reduced function devices (RFD) should be calculated in more detail in the case of the FFD dying due to its running out of energy and the sub-network having to reserve other FFD as an alternative cluster header. Additionally, this scheme did not support the DAD mechanism. This leads to serious problems regarding confliction if a node has a demand to communicate with others.

### 3.4. Role Configuration

Besides normal sensing nodes, other nodes play different important roles in WSNs. For instance, in coverage issues, due to power-constraints, sensors should switch between the on and off modes in order to save energy. In clustering, nodes are configured to achieve scalability, reduce overhead, and easily manage the network. Therefore, some nodes are assigned as the gateway, while others are assigned as cluster heads or assigned as normal nodes. Finally, in the in-network aggregation network, in order to save energy and reduce useless information, some nodes are configured as aggregators, sinks, or source nodes. Obviously, a mechanism to assign to the role of each node is necessary. However, again, the number of nodes is a significant concern.

**Figure 11:**
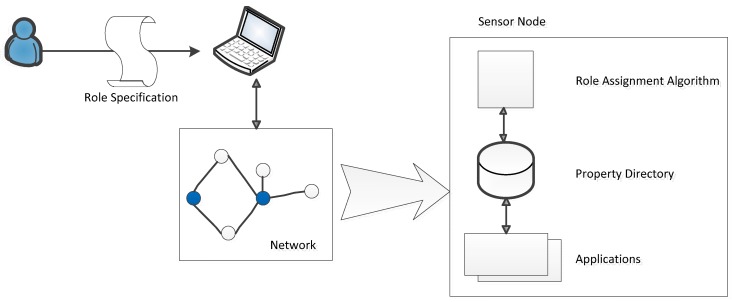
Core elements of the generic role assignment.

The process of role allocation is divided into a series of steps and is presented in detail in [19,20,21,22], as well as being shown in Figure 11. These approaches propose a role assignment framework that includes four parts: a set of role specifications that responds to a set of roles and assignment conditions, a role compiler that is able to translate the specification into an abstract representation at the network base station, the property directory at each sensor node that provides a flatform for each one, and the basic supplying services or applications.

Following the above framework, there are two steps required in setting up a role for a sensor: examining the role of a node and distributing those roles to a required node. The former one is implemented by a sequence of operations. First is defining a set of some pre-defined syntax and semantics. The final role of a node is achieved by performing AND and OR operations among the predicates. Each predicate is calculated through a simple predicate that uses Boolean operations. Count predicates, which count the nodes, satisfy some of the pre-conditions. The last classification is that of retrieve predicates, which are used to attach the matching nodes and the local property variable.

The second step is role distribution. There are several approaches to role distributions, which are presented below.

#### 3.4.1. Cache Algorithm

This algorithm is based on the local rule evaluation. There are three procedures: first, a node initializes a cache table. Next, the properties of that table are propagated to neighbors. Additionally, the last procedures require a node choosing a role, according to the local table.

**Figure 12:**
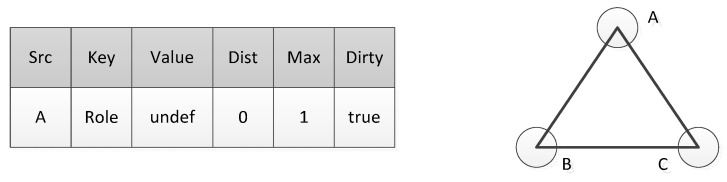
The role table and the relative topology of A, B, C.

In the first stage, all nodes share the same properties and form a table, as is illustrated in Figure 12. “Src” indicates the source node, which propagates the role-assigning message to this node. “Key” is the specific role. “Value” is the value of that role. “Dist” represents the hop-distance between the source and this node. “Max” decides whether the role-assigning message should be relayed to neighbor nodes or not. In addition, the “dirty” bit describes whether this information is delivered to neighbors or not. Therefore, there are only two values: true or false. The true is set if this bit is initiated or not delivered yet. In the next stage, a node decides to broadcast the information in the role table if (dist < max) and (dirty = true). Then, the dirty bit is set to false. Lastly, the local rule evaluation is scheduled.

#### 3.4.2. Probabilistic Initialization

Undefined initial roles, as presented in the above algorithm, may lead to inconsistencies of the initial configuration. To cope with this problem, a probabilistic initialization algorithm is proposed by choosing the initial role more carefully. The algorithm is as follows. First, a node starts with a set of specific roles. The probability p_r_ for the selection of each role should be estimated at each node. Then, the role is calculated based on the probability p_r_. This probability also can be calculated offline based on the static information. Next, a map between the role specification and a system of q equal to ins with q unknown is made. Lastly, the process of calculating the predicates, including count predicates, to retrieve the predicate is determined to be normal. However, instead of using each atomic predicate, this algorithm uses the probability of them.

#### 3.4.3. Wave Initialization

The above algorithms list a number of limitations, such as skipping the affection of the retrieved node ID over other predicates. The probabilistic cannot perform the node ID at the initial phase, which leads to additional interaction. Therefore, in order to overcome the above challenges and improve the stability of probabilistic decisions, conditional probabilities are used by leveraging the existing network flood.

Firstly, the update procedure begins from the sink to flood update packets to others. Each node receives that message and then waits for a random time, then chooses a role before forwarding that message with the chosen.

To examine the role of each node, once the node receives the update packet, it also applies the following scheme: calculate each predicate, count predicate, and retrieve predicate. However, in this context, the above processes are based on the role probabilities and roles of some nodes in that node’s scope.

#### 3.4.4. Heuristic Approach

This proposal is a combination of linear programming and genetic algorithms to assign specific roles for each sensor node in the optimal. The linear programming presents a way of minimizing energy consumption for data transmission between the nodes. In this context, a sensor network is arranged into a directed graph formed by a set of nodes and a set of arcs between nodes. The constraints are established by balancing the information flows, guaranteeing the active nodes, and capacity of each node. In addition, each node should play a role at that moment. Then, the genetic algorithm is used to distribute the role to the other nodes, based on the constrained, which is calculated by the integer linear programming.

The genetic algorithm is as follows: first, an initial population is generated in which each one is a potential solution. A fitness function or objective function is set by a sequence of mutation and regeneration processes. Then, the best result is found. However, it is important to note that when the number of nodes increases, the interval to achieve the optimal solution also increases and ultimately results in reducing the efficiency of the network.

### 3.5. QoS-Parameter Configuration

If addressing and assigning roles for sensor nodes is one of the critical problems in the first stage of configuration of a WSN, QoS-parameter allocation makes the WSN more efficient. Therefore, to meet this requirement, certain QoS parameters should be assigned for each sensor, such as the coverage range of a sensor. However, the existence of a set of parameters leads to a requirement of trading-off among those parameters. Various proposals [25,26,27] exist to discover the optimal solution for that problem. Table 3 presents an overview of some possible solutions.

#### 3.5.1. Flat Configuration

This scheme utilizes the evolutionary algorithms (EAs) to satisfy the trade-off between many configuration parameters. The whole process of calculating the optimal point of above trade-off is completed in two steps.

First, a WSN defines its own configuration space. Each network defines its parameters and the relationship between them. According to the protocol in [25], the parameters in each WSN are classified as global, which entails network-level parameters, and local, which entails node-level parameters. In addition, the whole network is vertically divided into multiple levels, such as node level, application level, network level, and MAC level. Parameters in each level can have either a positive or a negative relationship. Each node has a set of parameters. All the same kind of parameters are defined in a matrix wherein each node is represented as a point in the coordinators of the objective matrix.

Second, based on the above parameters, the genetic algorithms are applied to achieve the optimal solution. In the beginning, a number of fitness functions are defined, and an initial population of individuals is generated. A new generation is the result of genetic operations, which includes recombination and mutation. According to the Pareto optimal front, the best set of parameters will be found. If the output does not satisfy the fitness function, a new generation is mutated again.

This kind of method relies on the flat architecture, and, as a result, the scalability is limited to the small network. Similar to [26], this approach is near-optimal in order to meet a predictable time.

#### 3.5.2. Mapping

**Figure 13:**
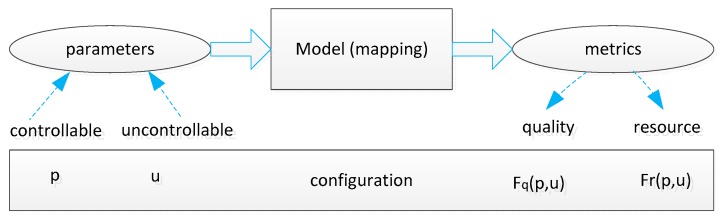
The basic mapping model.

To cope with the scalability problem, the proposal in [26] has solved the trade-off problem by proposing a mapping model in which the inputs are certain controllable or uncontrollable parameters, such as the lifetime of a node, and the output is the quality and resource metrics. After mapping the lifetime of a node, the reliability of a link, the power resource, etc., then, these metrics are optimized by finding the trade-off among them. The basic mapping model is shown in Figure 13.

**Figure 14:**
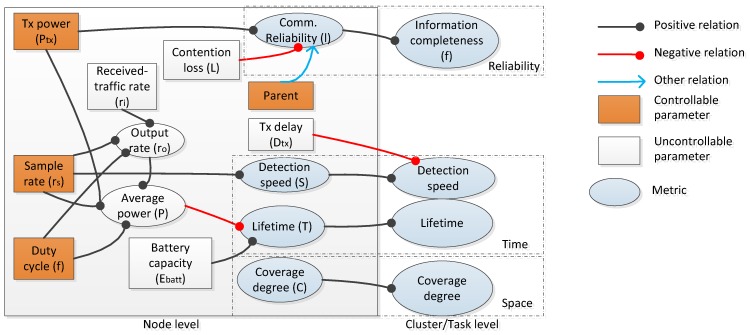
The hierarchical trade-off model.

The objective of the mapping model is to discover the relationship between the quality and resource metrics and the input parameters. Figure 14 illustrates a typical mapping model. In this figure, the input contains certain controllable parameters, such as power status and certain uncontrollable parameters, such as loss rate delay and battery capacity. Certain parameters have a positive or negative effect on others. Then, relying on the trade-off among them, some metrics are formed, such as communication completeness, detection speed, lifetime of a node, and coverage degree at the node level. Additionally, based on the hierarchical structure of the network, clustering topology, and the metrics at each node, the metrics at the cluster level are generated. That ends the first stage.

The next stage is the QoS-optimization phase utilizing the Pareto-optimal algorithm, which usually is applied in the flat network. In this context, in order to support the hierarchical network, first, this optimal algorithm is used for the node level. In other words, the lowest level is initialized to find the optimal point by using the Pareto-optimal WSN configuration. Later, the process of optimization is repeated in the larger network.

Obviously, this kind of approach is highly scalable and supports various parameters. This proposal also considers both controllable and unwanted parameters. However, if the number of the parameters increases, the relationship among them also increases, which leads to the difficulty of building the map, as is shown in Figure 14.

#### 3.5.3. Sub-Domain Based Configuration

**Figure 15:**
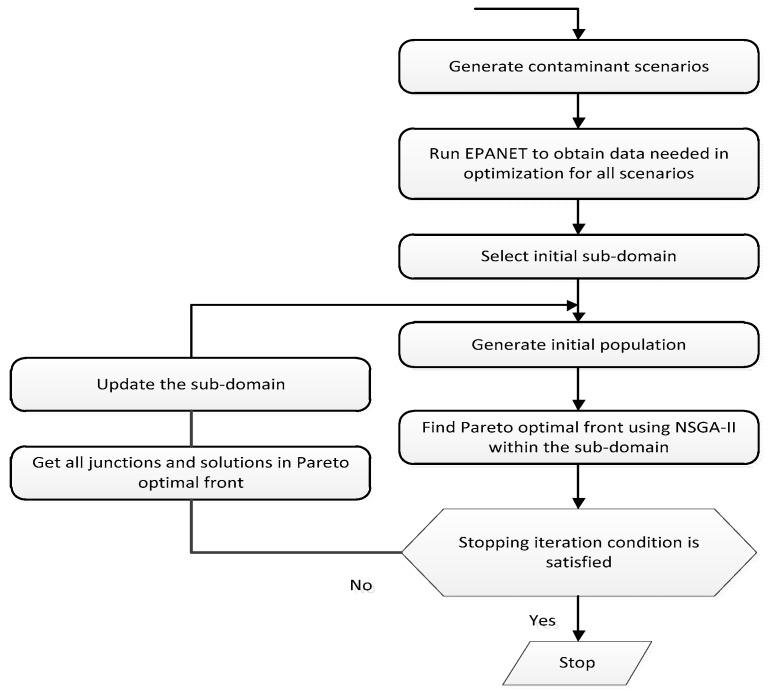
The flow chart of non-dominated sorting genetic algorithm-II (NSGA-II) processes (with permission from ASCE, adapted from [27]).

In order to reduce the complexity of the QoS assignment algorithm, this approach provides a simple method by taking advantage of the non-dominated sorting genetic algorithm and the Pareto algorithm to find out the optimal point of trade-off between configuring parameters. It is also a typical example when applying in an actual environment, the water distribution system. In the process of finding the best solution, first, some expected values are set as the objective functions of this problem. In this context, they are the minimizing of expected time of detection, the minimizing of expected water volume contaminated, and the maximizing of detection likelihood. The next step is described in Figure 15. The network is divided into multiple sub-domains. After collecting the necessary information, which should be optimized, and then choosing the sub-domain, a number of the initial population will be generated. Each member of the population contains a set of estimated parameters. By applying the non-dominated sorting genetic algorithm-II (NSGA-II) and the Pareto optimal front, the appropriate result is achieved if it satisfies certain pre-conditions. In other cases, the junctions and solutions will be obtained, and the sub-domain will move to the next step after updating its population. As is shown in Table 3, this method did not reach the optimal solution. Instead, it tries to satisfy some pre-requirements to meet time and energy constraints. Dividing the whole network into sub-domains helps to achieve a beneficial solution more quickly, which would make this algorithm scalable and adaptable to the large sensor network. However, this paper does not define how to form a sub-domain and how to choose a sub-domain.

## 4. Update Configuration

Sensors are required to be configured not only at the starting point, but also whenever there is a new requirement that should be delivered to each node. Then, beginning from the sink, the updating messages are broadcasted to all other nodes. These could be a new application, a parameter-changing or a new version of firmware, etc. They are different in terms of type, size, etc. The key idea of classification in this area is the amount of updating information to support the dissemination of this kind of packet. Therefore, our taxonomy is categorized into two approaches. In order to save energy, one is a minor update, which deals with small changes in configuration, such as changing parameters, while the other is a major update, which deals with some big substitutions, such as updating firmware.

### 4.1. Minor Update

As discussed above, a minor update is a general name for a set of strategy that copes with small changes in the configuration of sensors. Changing the value of a parameter is a typical example of a minor update. In this category, the authors tried to build a scheme that recognizes a small change, generate the update message, and then deliver that message to other nodes in the network. The biggest concern of this approach is how to create the updated message. Various schemes are presented below.

#### 4.1.1. TinyCubus

TinyCubus supports a mechanism that utilizes on-the-fly code update algorithms to disseminate software. A network consists of a set of inner nodes and a set of gateway nodes, with each node running an Operating System (OS) called TinyCubus OS. A new update is created with the support of the proposed TinyCubus OS. A generic framework is deployed in this OS to use for updating, which include three components: the Tiny data management framework, the Tiny cross-layer framework, and the Tiny configuration engine.

Besides the support of Tiny OS, an algorithm is presented for code distribution. According to this algorithm, first, the gateway starts broadcasting update packets to its k-hop neighborhood. These packets contain the roles for all of the nodes. Furthermore, once a node receives an update packet from a neighbor, it will send back an acknowledgment message to confirm. This ensures the success of the packet transmission.

This model deals well with the process of creation and dissemination of the update message. However, it does not have any mechanisms to take care of the process of activating the new configuration. By adding k parameters, the reliability of the configuring process is highly improved. A node can forward the update message to a k-hop node. However, if k is too large, it reduces the efficiency of the whole network. The implementation in [28] also indicates that by varying the number k of k-hop neighbors, the reliability and number of the control message for the successful delivering of each update packet increases. Although the above algorithm well supports updating, this is only applicable for a specific OS. If there are other kinds of OS, this method turns out to be impossible.

#### 4.1.2. Middleware

In order to reduce the intervention of humans, adapt quickly with the changes. If there are any new requirements, avoid reprogramming the source code and cope with the above challenge. Some proposals focus on building a middleware, which acts as an interface to interact with the application layer. Whenever there is a new update requirement from the application, it will be transformed in a special form through the middle layer and is then broadcasted to other layers. There are two existing proposals that have been presented about middleware [29,30].

#### 4.1.3. Ginconf

The proposal in [29] takes advantages of the middleware framework by proposing the GinConf model to adapt quickly with new requirements. In general architecture, GinConf is an abstract middle layer which consists of a set of components: I/O Adapter, which controls all traffic; acquisition, which relates to gathering data activities; data processor, which processes the streaming of data configuration; and API, which provides an interface for the previous components. All of these components are deployed in the application layer. Moreover, to support the industrial sensor networks, which consist of a large number of sensors, each of the components should be able to communicate with other nodes outside. This requires enabling the web server. There is also a server managing data traffic within a WSN to collect information and disseminate the update packets. Although this approach is deployed for a WSN in an industrial zone, it supports the web service to monitor the whole network, mechanism, and detail architecture for API. Which is actually responsible for adapting new update requirements is unclear.

#### 4.1.4. Context-Aware Middleware

**Figure 16:**
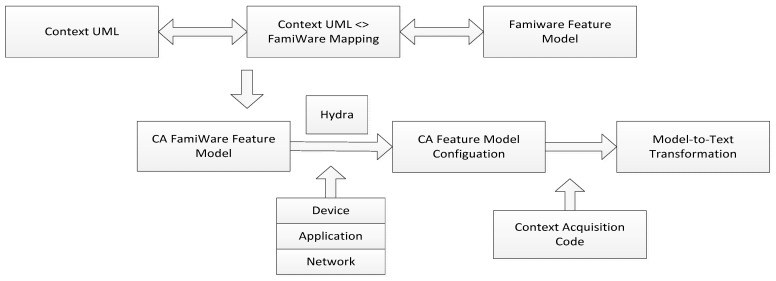
The illustration of FamiWare operations (adapted from [29,30]).

To overcome the limitation in [29], the proposal in [30] focuses on building a middleware named FamiWare to generate ready-to-install code, which supports a heterogeneous network, including WSN. This model uses the UML common language to express this requirement. Then, a map from the UML context to FamiWare is created. Next, the specific configuration of FamiWare is generated to cope with the requirement in the upper layer. Lastly, the FamiWare model automatically creates the code in every device in the system, as is demonstrated in Figure 16.

This kind of model supports for various kinds of devices, including sensors. Additionally, several different contexts are also applicable to this framework. After generating the specific configuration, depending on the kind of device, this information is delivered to each device. The distribution mechanism is followed by the publish/subscribe event-based mechanism.

However, due to the limitation of the functions and rules of the middleware, if there are any complex requirements, it could be impossible to deploy this mechanism for updating to other nodes. Therefore, in order to support a wide range of requirements, some proposals have tried to compare the source code in the source node and the destination node to discover the changes. Then, the changes are distributed to other nodes.

#### 4.1.5. Edit Script

Following the above scheme, the proposal in [31] has solved the update configuration problem by generating the edit script. This helps to reduce the number of update packets. According to [30], code distribution includes four processes: initialization, code image building, verification, and loading. However, two main processes are highlighted. The first one is optimizing the updated binary code. The additional process entails making the edit script. In the former task, after loading the new application, a series of operations are performed including inserting, copying, deleting, and repairing to optimize to code. In the further activities, these above operations are predefined by certain Opcodes. Then, the edit script is generated. In this context, the scripts in the source and the destination are compared to generate the differences. Then, the edit script generation algorithm is run to generate the edit script. The result in [31] indicates that by applying this method, the script size is dramatically reduced. This saves energy and memory for the updating process in case of resource-constrained in WSNs.

#### 4.1.6. Update-Conscious Compilation

Having the same goal with the approach in [31], the scheme in [32] provides a way to generate and update new requirements to remote nodes. This approach tried to avoid transmitting the similar code in update packets to save energy consumption. The new code is then generated in the form of a small script and is forwarded to the remote nodes.

The process of updating can be categorized into two steps:

First, an update script is created. At the sink, the original source code S and the source code after being updated to S are converted into the intermediate representation IR and IR’. Then, after the optimization process, IR and IR’ are compared and turned into binary code E and E’ by using the data allocation. Finally, E and E’ are compared to find out the different script U.

In the next step, the summarized script U is created and then is disseminated over the WSN. Some of the update methods are supported, such as copying, inserting, replacing, and removing. By running the script interpreter, a new binary image is generated and the update process is completed. The simulation in [31] shows that by using this scheme, the interval of update time has been reduced. Additionally, the overhead has also been reduced.

Table 4 summarizes classifications and comparisons of minor updated in WSNs.

### 4.2. Major Update

In contrast with the minor update, the major update is responsible for heavy, large amounts of update information. In other words, it can be the whole kernel image of the sensor network. Aware of the important role of this kind of update, most of the available mechanisms designed for this one are 100% reliable and scalable. Various proposals have been revealed as presented below.

#### 4.2.1. A Complete Model

The complete model [33] is a combination of three steps: generation, propagation, and activation.

Generation: first, a plan is made for a software update, so that the sink node estimates the necessary things, such as required power. Then, create the update packet, call the update tools, and be ready to advertise it.

Propagation: broadcast the update packets to neighbors with minimal transmission energy. Then, these packets will be relayed to the whole network. This step should take care of the loss packet. Therefore, once a node receives the packet, it will provide feedback to ensure a successful transmission.

Activation: this step occurs at the destination. Whenever the node completely receives the update packet, it decodes, checks the security, and tries to load the program file. Last, it must activate it and finish.

In this approach, due to many steps which include the feedback step, the speed for delivering a packet to a destination is often slow. However, the reliability is greatly improved. The feedback mechanism ensures that the packet will be successfully disseminated to all of the nodes.

Following this model, there are some detail schemes, such as MOAP, Mate, Impala/ZebraNet, and Deluge [34,35,36,37]. As the comparison among them in [33] indicates, the three first protocols seem to support most of the functions in the above model, while the last one only focuses on the propagation process. It ensures successful delivery of updating packets to all nodes in the network. However, all of them do not utilize the feedback and monitor mechanism. This may cause a loss of updating packets. Additionally, security should be considered in these approaches.

#### 4.2.2. RECOUP

To provide a lightweight mechanism, this protocol provides a procedure for highly reliable distributed configuration in a WSN. The process of updating consists of the flooding process and the local repair mechanism.

**Figure 17:**
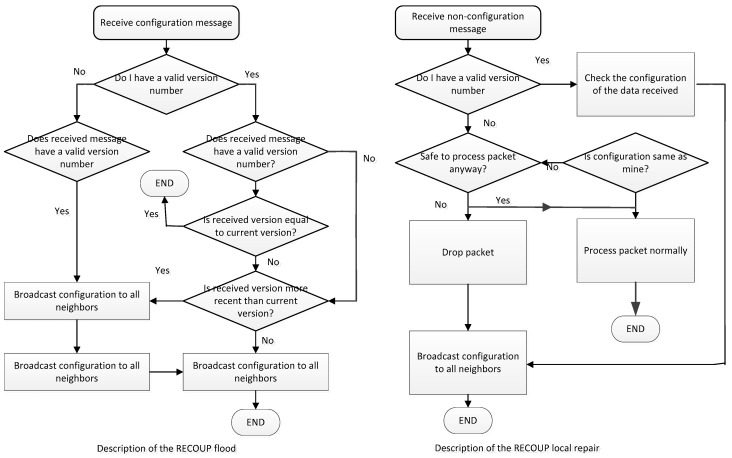
Flooding and local repair processes of the REGROUP algorithm.

Different from the above-mentioned schemes, this proposal focuses on how to reliably deliver the update packets to nodes in WSNs. Packets first are broadcasted throughout the network, as the left side of Figure 17 indicates. Then, if there are any errors, a local repair mechanism is proposed, as the right side of Figure 17 shows. In the former task, as is shown in Figure 17, whenever a new update is available, this will be flooded throughout the whole network. Once a node receives an update message, it checks the version number. If it is valid, the update processes as usual. Otherwise, the node is a new node and it needs to verify the current version. If the update version is more recent, this node will update. Additionally, in other cases, notification is used to provide information to all of the neighbors.

The local repair process is simpler. In this context, a node receives a non-configuration message. It then checks the version number and the safety of that packet to decide whether to update, as Figure 17 displays. After that, the difference between the received packets and the current version is checked to determine whether to update. The simulation results in [38] indicate that the ratio of the packet loss is reduced. This algorithm needs fewer control packets.

#### 4.2.3. A Simple Upgrade Method

To support a very simple updating mechanism, the algorithm in [39] is one of the good options. Once a sink wants to transmit an update to other nodes in the network, it just forwards this message to its relaying node. The next relaying node depends on the strategy. Three candidate strategies are proposed: Longest-Distance, Many-Neighbors, and Randomly-selected nodes. A node will choose the longest distance from the relay node in the Longest-Distance method, or will choose more than one neighbor in Many-Neighbors method, or will select a random node in the Randomly-selected node method. After receiving the packets, nodes change to upgrade mode and stop all sensing activity. Then, the update messages are relayed to others. This process is then repeated in others. This mechanism seems to be easy to implement. However, it does not consider the reliability problem. Most of the process focuses on how to deliver the packet and the state of the receiver. This kind of approach is difficult to apply without support from the other mechanisms.

Table 5 illustrates briefly the completion of the three above models:

## 5. Discussion

Lasting for a few decades, various approaches have been proposed to solve problems of auto-configuration in WSNs. However, still there are some open issues remaining. For instance, in the addressing problem [9,10,11,12,13,14,15,16,17,18], the hierarchical proposal seems to be scalable. However, these approaches require additional assignments, such as discerning different roles for different kinds of node. In addition, choosing a suitable node to become a cluster head is still a problem. Additionally, the header for each packet is bigger compared to that of the other approaches in this field, due to complexity of header. Each address includes the following information: the gateway’s address, the cluster header’s address, and the ID of that node. Then, this leads to requirements of the compressing header and packet. In contrast, the local addressing [14,15] approaches reduce the header of each packet. Moreover, an address for a sensor is assigned quickly. However, this kind of approach should only deploy for a small area where a node does not have any communication with others in an outside network. The ID for each node is unique to a specific area. If a node wants to communicate further, another mechanism should support that requirement. Additionally, this requires an efficiently-choosing server, due to depleting rapidly at the local server. From the above discussion, there is a trade-off between the scalability and addressing delay. Then, depending on the requirement of each network, an administrator should choose a suitable mechanism to deploy the address for this network.

The framework in [19,20,21,22,23,24] is useful for role assignment. However, the role calculated for each node is complex and independent. Similarly, in the QoS-parameter assignment, the trade-off among the parameters is solved by the Pareto optimal, which is a mathematical model and seems to be difficult to apply in the real world. This is because it requires a high capacity to calculate. Moreover, these schemes are centralized, and there is a demand on a light-weight distributed calculating mechanism to adapt with the dynamic condition of a WSN. In addition, the QoS value of a node, such as its power resource, affects the role of a node. Therefore, the node’s role assignment and QoS-parameter assignment [25,26,27] should be allocated together.

In the event of an update configuration issue [28,29,30,31,32,33,34,35,36,37,38,39], a middleware could adapt quickly with a new requirement. However, the range of requirement is restricted due to the limitation of supporting applications. Other approaches deal well with this problem by comparing the old and new versions and discovering the differences and then delivering them. However, once there is a new change implemented, a node has to execute the whole process to update. It costs energy and resources greater than that of the middleware approach. Then, depending on the deploying requirement, low updating delay, or a wide range of supporting applications, a suitable option would be chosen.

The framework in [33] is complete to update software and firmware. However, in implementation, some proposals [34,35,36,37] have not taken care of feedback nor monitored the update process. This could lead to a potential loss of packets while updating. Moreover, there is no clear scheme for providing a feedback mechanism in that framework. Although the later mechanism occurs in a major update, they cannot guarantee the reliability for the updating process. In this case, reliability is the highest priority and the framework in [33] is the most suitable one.

Network management includes the process of managing, monitoring, and controlling a network. Existing management protocols for wireless sensor networks focus on how to collect the network status and use the information to manage the network [51,52]. MANNA [53] performs centralized fault detection based on analysis of gathered WSN data. However, MANNA provides no automatic network reconfiguration to allow the network to recover from faults and failures. sNMP [54] proposes a network management framework with two functions. The first function is to define sensor models that represent the current state of the network. The second function is to provide algorithms and tools for retrieving network state through the execution of the network management functions. In SNMS [55], the authors propose an interactive system for monitoring the health of sensor networks. SNMS implements two management functions with query-based network health data collection and event logging. The key advantage of SNMS is that it introduces overhead only for human queries and minimal impact on memory and network traffic. The limitation of SNMS is that the network management feature is limited to passive monitoring only, so it requires human managers to submit queries and perform analysis of management data. Overall, the existing network management protocols do not provide auto-configuration functions. In our opinion, integrating auto-configuration schemes to network management protocols is necessary to have efficient and completed network management tools. For example, adding the auto-configuration function to MANA may allow the network to recover from faults and failures automatically. In addition, network status monitoring and topology discovery functions of the network management protocols may also help auto-configuration operations to be executed faster and more energy-efficient.

In [56], the authors present a novel approach for the joint implementation of energy prediction and transient computing aimed at improving the adaptability and robustness of applications running on battery-less nodes. In [57], autonomous network techniques are applied to the building environment and energy monitoring (BEEM) to improve the transmission reliability of the radio frequency technology with light energy harvesting featured power supply and energy management system. Similarly, in [58], autonomous network techniques are proposed to integrate a novel RF energy harvesting circuit which gathers the energy from an 868 MHz RF signal source for remote powering. In [59], autonomous techniques are used for WSN deployment by using mobile robots. The existing autonomous WSN studies are more likely about adapting or self-adjusting parameters at the node level and are not directly designed for auto-configuration. In our opinion, autonomous network techniques can be useful for WSN auto-configuration and WSN auto-configuration can also be helpful to complete autonomous network implementation. Therefore, integrating auto-configuration into autonomous network implementation for WSNs is an interesting topic for future work.

## 6. Conclusions

This paper presents a comprehensive understanding of recently-proposed auto-configuration schemes in WSNs. The important challenge that all proposals in this paper deal with is efficiently configuring for a large number of nodes in WSNs with various parameters. For additional information configuration, group-based addressing achieves a better energy efficiency in comparison with geometric-based and location-based addressing, but the unique characteristic is the remaining issue. For QoS parameter configuration, the mapping approach provides a more scalable and optimal solution, but the complexity is the remaining issue. On the other hand, the sub-domain based configuration approach provides a lower complexity solution but scalability is the remaining issue. For the minor update, the comparison shows that the middle layer approach provides low overhead and high speed of adaptation. This approach has advantages in comparison with other approaches when applying to resource-constrained sensors. For the major update, existing approaches show clear tradeoffs in terms of energy efficiency, completion time, and complexity. More investigation in this configuration type is expected to achieve a good tradeoff between the complexity and completion time. From the above discussion, we can conclude that solving the trade-off between the scalability and addressing delay is important for choosing a proper addressing approach. Next, although role assigning and QoS-parameter assigning schemes solve their own problems well, they should be combined in order to save energy, and also due to co-existing between two kinds of parameters. In an updating problem, the trade-off between a low-updating delay and a wide range of supporting applications determine the chosen approach. Whenever there is an upgrading demand, the framework in [33] is a good choice if it is fully supported.

## Figures and Tables

**Table 1:** Summary of the address auto-configuration.

Method	Hierarchy	Unique Address	DAD	Distributed	Address Space	Overhead	Latency	Scalability
Proxy-based addressing	Yes	Yes	Yes	Yes	16 bit	Low	Low	Yes
Multi-tier	Yes	Yes	No	Yes	Vary	Large	Low	Yes
Cluster-based	Yes	Yes	Passive	No	128 bit	Large	Low	Yes
Localized	No	No	Yes	No	Vary	Low		No

**Table 2:** Summary of configuration mechanisms with additional information for WSNs.

Method	Scalability	Energy Efficient	Address Unique	Address Space	Structure	DAD
Group-addressing	Yes	Yes	No	Vary	Hierarchical	Yes
Geometric-based addressing	Yes	No	Yes	16 bits	Hierarchical	Yes
Location-based addressing	Yes	No	Yes	128 bits	Hierarchical	No

**Table 3:** Overview of QoS-parameter configuration in WSNs.

Method	Scalable	Scope	Optimal	Hierarchical	Method	Complexity
Mapping	Yes	Any	Yes	Yes	Pareto optimal front	High
Flat configuration	No	Not large	Near	No	Dominated sorting algorithm, Pareto optimal front	High
Sub-domain based configuration	Weak	Any	Near	Yes	Pareto optimal front, NSGA-II	Low

**Table 4:** Classification of minor updates in WSNs.

Method	Reliable	Update Time	Overhead	Repair	Speed of Adaptation
TinyCubus	Low	Long	High	No	Low
Middle layer	Low	Short	Low	No	High
Efficient Code Distribution	Low	Short	Low	No	Low
Update-conscious	Low	Long	Low	No	Low

**Table 5:** Overview of existing approaches in major updating.

Model	Reliable	Update Time	Repair	Energy Efficient	Completion of Process	Security	Complexity
Complete model	High	Long	Yes	Low	Complete	Yes	High
RECOUP	High	Short	Yes	Medium	Focus on distributing update packet	No	Medium
A simple upgrade method	Low	Short	No	Medium	Lack of most process.	no	Low
